# Superposition of Transcriptional Behaviors Determines Gene State

**DOI:** 10.1371/journal.pone.0002901

**Published:** 2008-08-06

**Authors:** Sol Efroni, Liran Carmel, Carl G. Schaefer, Kenneth H. Buetow

**Affiliations:** 1 Center for Biomedical Informatics and Information Technology, National Cancer Institute, National Institutes of Health, Rockville, Maryland, United States of America; 2 National Center for Biotechnology Information, National Library of Medicine, National Institutes of Health, Bethesda, Maryland, United States of America; 3 Laboratory for Population Genetics, National Cancer Institute, National Institutes of Health, Bethesda, Maryland, United States of America; University of Arkansas for Medical Sciences, United States of America

## Abstract

We introduce a novel technique to determine the expression state of a gene from quantitative information measuring its expression. Adopting a productive abstraction from current thinking in molecular biology, we consider two expression states for a gene - *Up* or *Down*. We determine this state by using a statistical model that assumes the data behaves as a combination of two biological distributions. Given a cohort of hybridizations, our algorithm predicts, for the single reading, the probability of each gene's being in an *Up* or a *Down* state in each hybridization. Using a series of publicly available gene expression data sets, we demonstrate that our algorithm outperforms the prevalent algorithm. We also show that our algorithm can be used in conjunction with expression adjustment techniques to produce a more biologically sound gene-state call. The technique we present here enables a routine update, where the continuously evolving expression level adjustments feed into gene-state calculations. The technique can be applied in almost any multi-sample gene expression experiment, and holds equal promise for protein abundance experiments.

## Introduction

In examining genes, either individually or in system-wide characterizations, it is useful to generalize its “state”. For example, a gene's *Present/Absent* call is a common dimension of the reported results of gene-expression microarray experiments. Such calls tag each probe set in the microarray with a determination of whether the probe set is expressed (Present) or unexpressed (Absent) in the sampled tissue [1]., *Present/Absent* calls are often used in filtering out false positives from the large collection of probes on an expression array. The most commonly used approach to making such calls is the MAS5 algorithm [1], part of the Affymetrix™ collection of software tools [2]. While some recent experimental findings support the use of the MAS5 algorithm [3], MAS5 has some significant shortcomings. First, MAS5 does not provide the user with a statistical gauge of the basic claim behind the *Present/Absent* call. Second, MAS5 does not compare calls across multiple samples. Finally, because MAS5 does not operate on adjusted readings, it cannot benefit from the increasingly sophisticated techniques for adjusting gene expression readings (e.g. RMA [4] and others [5]; see [6] for a comparison of techniques)

Conceptually it is understood that the classification of genes into alternative states is a simplification of much greater complexity patterns of gene behaviour and action. However, empiric evaluation of the observed data finds that gene expression patterns commonly can fit one of two alternative expression level distributions. Moreover, such simplification has proven valuable in other research domains. For example the simplification that abstracts digital logic from the underlying continuous flow of electrons in integrated circuits has enabled the design of devices of staggeringly complex functionality [7].

We describe here a method that makes use of quantitative expression level readings. It is important to stress that the method is not a pre-processing step, like background adjustment for noise, but rather a post-processing step that makes use of the noise-adjusted readings. In the specific examples presented here, we make use of RMA-adjusted expression levels [4] from Affymetrix microarrays, but the input could be raw or adjusted values from any platform. Using the expression levels, we build a statistical model of expression for each probe set, based on an assumed bimodal distribution, that accounts for the two states of an expressed gene: Up and Down.

**Figure 1:**
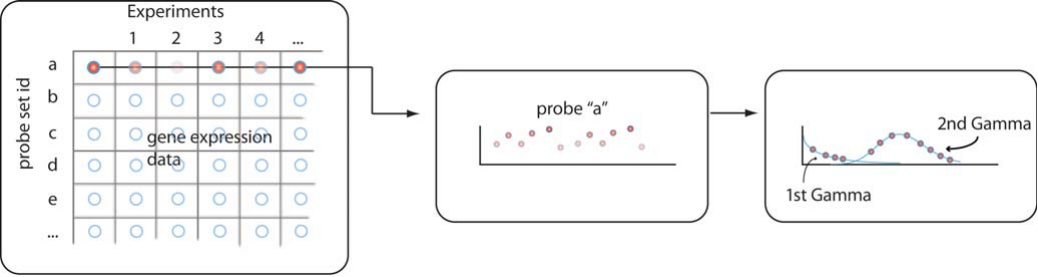
Data handling in the mixture algorithm. First, gene expression data from a set of gene expression experiments is collected. The matrix in the figure shows rows probe set, where every row is a single probe set, and every column is a different hybridization experiment. This could be, for example, Affymetrix microarray experiments, where each column is a different patient. We then look at the data probe-by-probe. For example, we follow probe “a” in the figure and look at the expression levels for this probe, across all samples in the set of gene expression experiments. Each probe will have data from the entire collection of experiments. For the specific probe “a”, we fit the set of expression measurements into two gamma distributions, one representing the “down” state and one representing the “up” state. Each data point is then computationally associated with a probability of being either under the first Gamma distribution (which would mean the gene associated with the probe, for the specific sample, is at a “down” state) or with the second Gamma distribution (which would mean the gene associated with the probe, for the specific sample, is at an “up” state). We iterate the procedure across the entire probe-set, to tag every gene across the microarray with its probability of being “up” or “down”.

The inputs to the statistical model are the probe-set expression levels from multi-sample experiments. For a specific probe set, we gather expression levels from the cohort of samples for. For example, in a set of experiment involving 100 patient samples and 100 control samples, we obtain 200 data points for the single probe set (see Fig. 1). We then use the data points from the single probe set to infer two gamma distributions, one distribution representing the *Down* state and one representing the *Up* state. Our choice of gamma distributions comes from the distribution flexibility in containing the two distribution shapes we required. Such mixture models have been successfully applied to other problems in biology (e,g, [8]–[10]).

A gamma distribution has the general form:
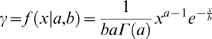
where *a* is often called the shape parameter and *b* the scaling parameter. The general form of a gamma distribution is beyond the scope of this paper (see [11] as a reference). For smaller values of *a*, the gamma distribution takes an exponential-like form, with a continuous decay that starts at zero; for larger values of *a*, the distribution takes a form similar to the normal distribution, with a mean of *ab* and variance of *a*−*b* ^2^.

By combining single probe data across multiple samples, we consider the entire population of probe expression values (gene values) as derived from a single distribution. That single distribution is in fact the mixture of two gamma distributions – one distribution for the Up state and one distribution for the Down state. We represent the resulting model with six parameters: *a_u_*, the shape parameter for the Up distribution; *b_u_*, the scale parameter for the Up distribution; *a_d_*, the shape parameter for the Down distribution; *b_d_*, the scale parameter for the Down distribution, and *η_u_*, *η_d_*, the mixture coefficients that give the relation between the two distributions in the final mixture. We determine values for the different parameters using an Expectation-Maximization (EM) algorithm (see Methods), the output of which are the six defining parameters. Upon completion of processing using the gamma mixture (GM) algorithm, we are able to calculate, given a specific expression value, the probability this expression value represents a gene in the *Up* (or *Down*) state.

## Results

To compare the consistency of MAS5 calls with the consistency of GM calls, we used the publicly available results of a spike-in experiment [5]. In this experiment, the researchers assayed samples that were identical except for controlled differences in the RNA of 42 transcripts. Except for the 42 transcripts whose levels were systematically manipulated, each probe would be expected to have the same *Present/Absent* call across the experiment and to have the same *Up/Down* call across the experiment. We measure the success of the two algorithms by their consistency over the cohort of sample. A perfect score for an algorithm would mean that the algorithm succeeded in finding identical *Present/Absent* or *Up/Down* call for each of the genes across the experiment.

Of the 22,283 probes examined in the experiment, the MAS5 algorithm was consistent in assigning the same *Present/Absent* call, across all samples, for each of 17,004 probes; the remaining 5278 probes were assigned inconsistent calls by MAS5. In contrast, the GM algorithm consistently assigned the same *Up/Down* call for each of 19,923 probes and gave inconsistent calls for the remaining 2359 probes. Thus the GM algorithm showed an improvement of 55% in consistency.

**Figure 2:**
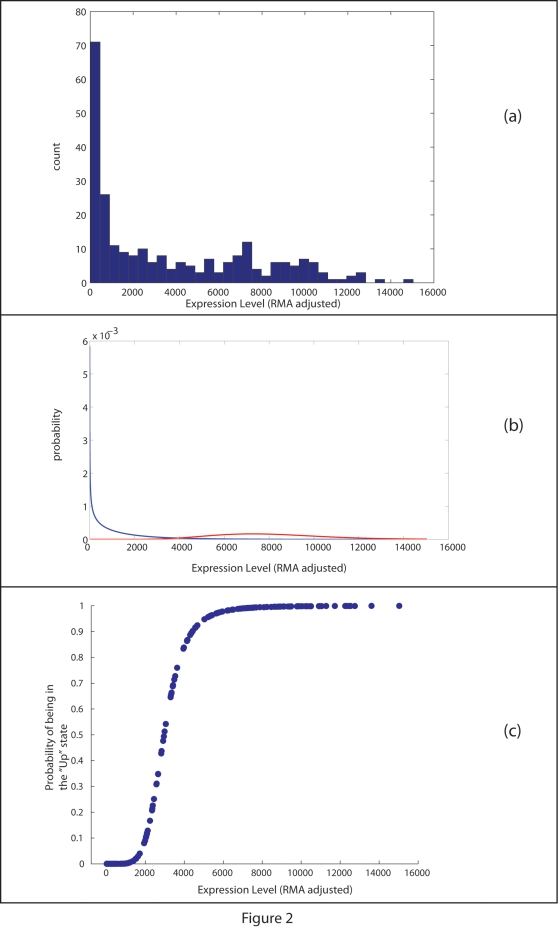
An in-depth look into data from a single probe over a collection of 251 hybridizations of breast cancer samples. (a) Displays a histogram of gene expression data for a single probe across the collection of 251 samples. The x-axis corresponds with levels of expression and the y-axis is a count of expression level for the specific bin. As the panel shows, many of the hybridizations show a level of expression close to zero. This is visible through a large collection (large count) of gene expression measurements close to zero expression levels. On the other hand, many of the probes show expression levels that spread across the entire span of expression levels from zero to 16,000. This is well-fitted into a gamma mixture model that assumed a dual behavior for the gene. Panel (b) shows a plot of the approximated distribution functions across the entire gene expression range for the surveyed probe. The two lines plotted have been calculated by fitting gene expression into the assumption of a binary state distribution, with each distribution modeled by a Gamma-like behavior. Panel (c) gives the probability of being in an “up” state as a function of gene expression for the specific probe surveyed. As panels (b) and (c) overlap, we demonstrate how changes in expression levels in (c) associate that level with the curves of panel (b). The higher an expression level in (c) is, the more probable it is to be affiliated with the red curve (“up” curve) of panel (b). The lower the expression level is in (c), it is more probable to affiliate it with the blue curve (“down” curve) of panel (b), and with a “down” state. Being in a “down” state is the reciprocal of being in an “up” state, which gives a probability of zero for being “up”.

**Figure 3:**
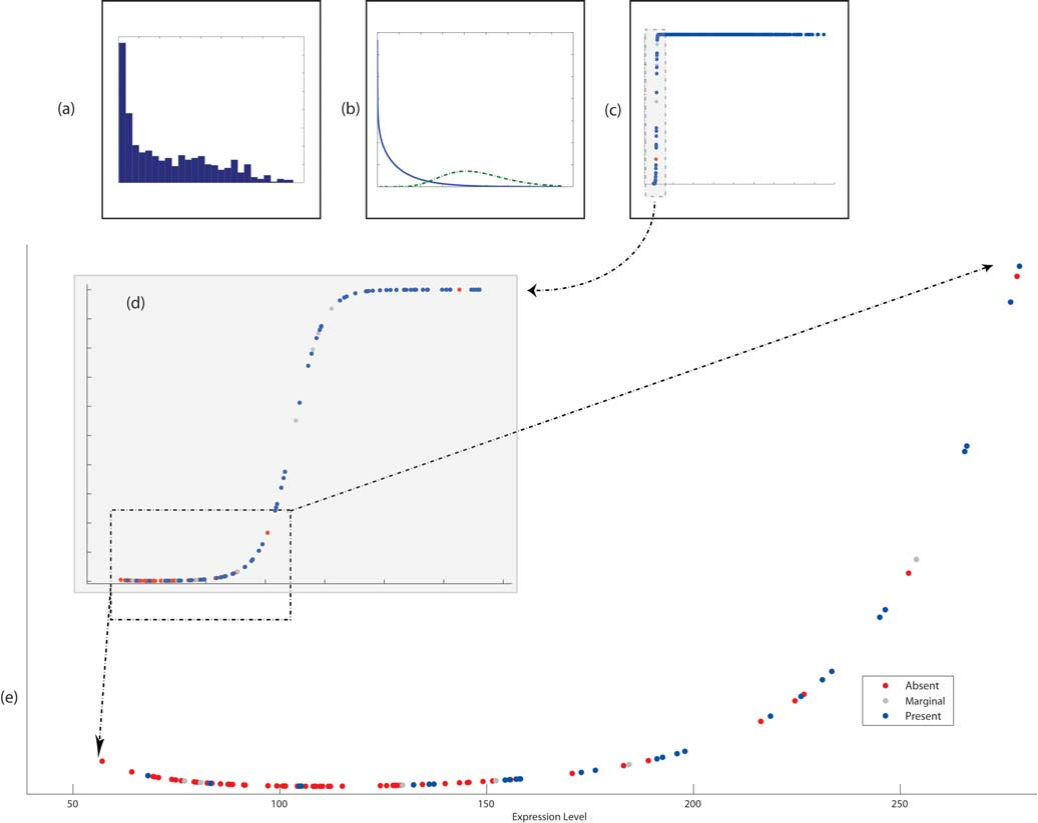
Similar to matching panels (a) (b) and (c) in Figure 2, panels (a), (b) and (c) of this figure show the histogram of gene expression, two resulting gamma curves, and the probability of being in an “up” state for a specific single probe set out of the collection of probes set in a collection of sample. Panel (d) is a zoom into the highlighted part of panel (c). Panel (e) is a zoom into the highlighted part of panel (d). Panel (d) shows the gradual probabilistic transition from being associated with a “down” state to being in an “up” state. The transition correlates well with gene expression and demonstrates the sensitivity of the approach to changes in gene expression. In panel (d) and in zoom-in panel (e), we also highlight the Present/Absent calls made by MAS5. Especially in the panel (e), it is easy to see how MAS5 Present/Absent calls toggle with growing levels of expression, despite an expected plateau, the MAS5 algorithm stabilizes on a Present call at much higher levels of expression and makes an Absent call for gene expression level as high as 500, while giving a Present call to expression level of 50. Use the toggled calls puts the user in danger of associating very different of expression level with very different states of a gene.

To examine the performance of the algorithms on data with natural biological variation, we turned to other publicly available studies. One such study, Miller et. al. [12], provides U133-A/B data on 251 primary invasive breast tumor samples. We are especially interested in the ability of the MAS5 and GM algorithms to make calls that are consistent with (RMA-adjusted) expression levels. That is, we expect an *Absent* or *Down* call to correlate with low levels of expression and a *Present* or *Up* call to correlate with high readings. Figure 2 shows an example, probe set ‘206378_at’ (which represents the gene SCGB2A2), where these expectations are confounded. Panel (a) shows a simple histogram of expression levels from the probe set, across all samples; Panel (b) shows the derived probability distribution, based on the Gamma Mixture hypothesis; and Panel (c) plots the probability of being in an *Up* state, as a function of the expression level. As the figure shows, the *Up/Down* classifications produced by GM algorithm correlate well with expression values, across the range of expression values. The MAS5 algorithm, on the other hand, toggles between *Present/Absent* calls quite sporadically in the expression range. To compare the MAS5 calls and the GM calculated probability over a large set of samples, we made use of data from [13], following the procedure described in Methods to obtain *Present/Absent* and *Up/Down* readings. Figure 3 shows the different readings. Panel (a), (b) and (c), as before, show the expression distributions of the probe set. In panel (d) a zoom-in view of the transition area of panel(c), shows the details of decision of the shift between the *Down* and *Up* status. In Panel (e), we can see the differences between decision based on the GM algorithm and the MAS5 algorithm, where low levels of expression values are toggled between present and absent calls made by MAS5 and, on the other hand, have low probability to be in the Up state (or high probability to be in the Down state).

## Discussion

We have described a new approach to determining the expression state of a gene. Like the Affymetrix MAS5 algorithm, our method is a two-state classifier. In contrast to the MAS5 algorithm, our method takes account of the underlying distribution of expression values in a set of samples. In particular, our method assumes a two-state distribution of gene expression that can be captured by mixed gamma distributions. As we have shown, our technique yields more stable calls than MAS5. In a set of biological replicates, MAS5 produced inconsistent calls for twice as many probe sets as the GM algorithm. Further, in a set of samples showing normal biological variation, the GM algorithm yielded calls that had better correlations with RMA-adjusted expression levels than the MAS5 calls.

Table 1 shows the strengths and weaknesses of the two approaches. One of the main differences – both a weakness and a strength – is the fact that the MAS5 algorithm is applied to individual samples, while the GM algorithm is applied to a set of samples. On the one hand, this allows MAS5 to be applied to individual samples from arbitrary experiments; on the other hand, MAS5 cannot take advantage of the statistical power in the multi-sample joined population. Second, the GM approach is not limited to readings from Affymetrix platforms, but may be applied to any values that represent gene expression or protein abundance. Third, since MAS5 is applied to raw values, it cannot take advantage of new adjustment techniques such as RMA; in contrast, the GM algorithm can be applied to raw or adjusted values. . Fourth, while MAS5 does give a p-value for the Present/Absent decision, this p-value cannot be interpreted as a distance from population and does not convey biological information.

**Table 1:** 

Technique	Potential use	Ability to incorporate new adjustment techniques	Statistical significance to results	Platform dependent
MAS5	Affymetrix-based. Can be used on a single samples.	No	p-value	Yes
GM (gamma mixture)	Any gene expression reading. Can be used only in multi-sample experiments	Yes.	Yes	No

In summary, we believe our approach to be a general and powerful way to fit gene expression data to a two-state model. We consider the GM call to be a true, scale free, normalization that is entirely platform-independent, applicable to any gene expression. While applied to gene expression microarrays that measure RNA abundance, this method is applicable to any quantitative measure of individual gene state.

## Methods

### EM algorithm

For each probed gene, the algorithm determine six parameters that define, together, the coefficients for each of the distribution (Up, Down) and the mixture coefficients between the two distributions. We call the set of different parameters *θ*

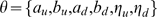
Where *a_u_*, *b_u_* determine the coefficient of the Gamma distribution that describes the Up gene state; *a_d_*, *b_d_* determine the coefficients of the Down state and *η_u_*, *η_d_* determine the mixture coefficient (and *η_u_*, +*η_d_* = 1).

The algorithm is iterates over the different function, so that every iteration improves the estimate of the coefficients. In [14] you can see the general proof of the EM algorithm, according to which, it is sufficient to find maximas for the function *Q*, defined as:
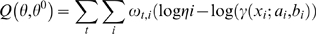
Where *θ* is defined previously as the collection of parameters. *θ* ^0^ stands for the set of parameters at a previous iteration and the index *i* goes over the two different function in the mixture and the index *t* goes over available data points.
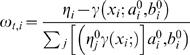
Finding maximas of *Q* replaces (the harder task of) finding maximas for the original function. To find maximas for *Q*, we differentiate it with respect to the model parameters and compare to zero. First according to *b_i_*:
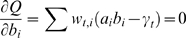


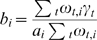
And then according to *a_i_*:
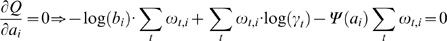
Where Ψ(*x*) is the psi function 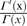.

Using a Lagrange multiplier to incorporate the constrain
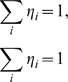
we have to maximize the target function
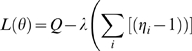
with respect to the *η_i_*, we derive
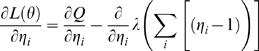
and obtain
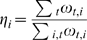
We solve this numerically (using Matlab®) in every iterative step, until we reach some predefined convergence criterion

### Gene expression spike-in data

We used Affymetrix's deposited gene expression data for a SpikeIn experiment, as it is available from [5].

Adjustments of the data were made using the RMAExpress tool [15] over original CEL files. Affymetrix's Present/Absent call (MAS5 calls) were made using Affymetrix's GCOS tools [1].

Other sources of gene expression data:

Data for the set of Bittner et. al were made available from data made publicly available by the Expression Project for Oncology, an International Genomics Consortium public/private initiative [13]. Data from Miller el. al [12] has been obtained from the Gene Expression Omnibus [16].
